# Development of *Stegasta bosqueella* (Chambers) (Lepidoptera: Gelechiidae) in Peanut Cultivars

**DOI:** 10.1007/s13744-026-01435-1

**Published:** 2026-09-11

**Authors:** Mirella Marconato Di Bello, Bruno Henrique Sardinha de Souza, Eduardo Neves Costa, Luciano Nogueira, Carlos Alessandro de Freitas, Bruna Ferrari Schedenffeldt, Marcelo Mueller de Freitas, Elias Silva de Medeiros, Arlindo Leal Boiça Júnior

**Affiliations:** 1 Depto de Ciências da Produção Agrícola, Univ Estadual Paulista (UNESP), Jaboticabal, SP Brazil; 2 Depto de Entomologia, Univ Federal de Lavras (UFLA), Lavras, MG Brazil; 3 Depto de Proteção Vegetal, Univ Estadual Paulista (UNESP), Botucatu, SP Brazil; 4 Instituto Federal de Educação, Tecnologia Goiano (IF Goiano), Posse, GO Brazil; 5 Centro de Ciências da Natureza, Univ Federal de São Carlos, Buri, SP Brazil; 6 Univ Federal da Grande Dourados (UFGD), Dourados, MS Brazil

**Keywords:** Antibiosis, *Arachis hypogaea*, Constitutive resistance, Host plant resistance, Integrated pest management

## Abstract

*Stegasta bosqueella* (Chambers) (Lepidoptera: Gelechiidae) is a major peanut pest (*Arachis hypogeae* L.) in Brazil. The evaluation of peanut cultivars is fundamental for characterizing the levels of insect resistance, supporting their use in integrated pest management (IPM). This study evaluated possible sources of antibiosis-type resistance to *S. bosqueella* in selected peanut cultivars*.* Five cultivars were evaluated for constitutive resistance levels: IAC Caiapó, IAC Runner 886, IAC OL3, IAC 503, and Granoleico. The development of *S. bosqueella* was evaluated under laboratory conditions, where neonate larvae were kept in Petri dishes and fed leaves of the peanut cultivars until pupation. The evaluated biological parameters of *S. bosqueella* were larval and pupal weights; larval, prepupal, and pupal periods; adult longevity; sex ratio; and larval survival. Larvae fed leaves of the IAC 503 cultivar presented prolonged larval periods, lower weights and survival. In addition, larvae fed on this cultivar gave rise to pupae with lower weights, adults with shorter longevity, and those with the lowest fitness index. Thus, IAC 503 was classified as resistant to *S. bosqueella* through antibiosis, whereas the other cultivars presented different levels of susceptibility. The results obtained herein can support the use of resistant cultivars in IPM for peanut crops since IAC 503 is a commercial cultivar that is readily available for planting by farmers. Additionally, IAC 503 can be useful in peanut breeding programs targeting *S. bosqueella* resistance, serving as a resistance gene donor.

**Supplementary Information:**

The online version contains supplementary material available at https://doi.org/10.1007/s13744-026-01435-1.

## Introduction

Peanuts (*Arachis hypogeae* L.; Fabales: Fabaceae) are cultivated in more than 100 countries and are considered the fourth most cultivated oilseed plant species in the world, with a production of 50,436 million tons in the 2022 harvest (USDA 2023). Peanuts stand out among annual crops because of their use in human food consumption and as raw materials for industrialized products, such as oil extraction, in the canned food industry, and in medicinal products (Rodrigues et al. 2016). China, India, the United States, and Nigeria are the world's leading peanut producers, whereas Brazil ranks among the major peanut-producing countries. According to the National Supply Company (CONAB), Brazilian peanut production reached approximately 1.18 million tons in the 2024/2025 growing season (CONAB 2026).

Insect pests can affect crop establishment, plant growth, and productivity, causing economic losses to farmers. More than 50 insect species have been reported to cause injury to peanut plants, among which thrips, caterpillars, and mites stand out (Smith Jr. and Barfield 1982; Almeida 2015; Danso et al. 2024). The rednecked peanut worm (RPW), *Stegasta bosqueella* (Chambers) (Lepidoptera: Gelechiidae), is a primary peanut pest in South America (Pinto et al. 2020a; 2020b). This is due to the infestation intensity and extent of damage that occurs throughout the peanut crop cycle (Boiça Júnior et al. 2012).

When the young leaflets of RPW-infested peanut plants open, they present symmetrical lesions, a characteristic of pest injury (Janini 2011). RPW attacks the shoots when the leaflets are still closed or even the buds; as the larvae chew these plant structures, the leaf area is reduced, in addition to preventing the opening of leaflets, which delays plant vegetative growth (Almeida 2013). At high population density, RPW can occupy the terminals of leaflets, causing destruction of the meristematic tissue and further reducing the number of leaflets, which can prevent plant growth completely (Wall and Berberet 1979), resulting in up to 65% yield reduction (Scarpellini and Busoli 2001).

The main control method of RPW in peanut crops consists of the use of synthetic insecticides. The products registered to control RPW in peanut crops are chemical groups of pyrethroids, neonicotinoids, benzoylureas, and organophosphates (Agrofit 2025). The use of contact insecticides has shown little effect since the larvae shelter between closed leaflets, thus being protected from spraying (Galli and Arruda 1989). To achieve full success in the use of chemicals, the cost‒benefit ratio must be determined, as well as environmental safety (Almeida 2013). Another technique that is sometimes incorporated into *S. bosqueella* management is the use of food attractants to monitor pest incidence (Rivero et al. 2017).

Host plant resistance is an important control tactic in integrated pest management and can help keep pest populations below economic injury levels in crop plants (Stout 2013). In addition to this benefit, the use of resistant cultivars has little or no negative impact on agroecosystems, does not increase the production cost related to pest control, and allows compatible integration with other IPM control methods (Smith 2005; Boiça Júnior et al. 2013).

The resistance of plants to insects can be expressed as antibiosis when the overall negative effects impact the biological aspects of the insect, such as reductions in larval and pupal weights, survival rates, extension of developmental periods, and alterations in adult fecundity and fertility, and antixenosis is expressed as defensive plant traits that affect the behavior of insects, preventing or impairing their feeding or oviposition, ultimately reducing pest colonization on host plants. The other plant defense category known is tolerance, which is classified as a resistance type by some authors (Smith & Clement 2012; Souza 2025) and as a distinct defense strategy of plants by others (Stout 2013; Mitchell et al. 2016). This difference is because tolerance traits do not negatively impact insects and are related to plant compensatory growth, reallocation of photoasymilates to plant organs and structures, and antioxidant metabolism, among other factors, preventing economic damage to support insect herbivory (Peterson et al. 2017).

In this work, we explored only the antibiosis resistance of peanut cultivars, evaluating the effects on RPW development. The evaluated cultivars were selected from previous screens via preference feeding assays with RPW larvae (Di Bello et al. 2015). The identification of peanut genetic materials with traits of resistance to insect pests is highly important for IPM and plant breeding programs (Michelotto et al. 2017). In Brazil, few studies related to the resistance of peanut cultivars to insect pests exist. Previous studies have shown that some peanut genotypes are less preferred for feeding by *Spodoptera cosmioides* (Walker) (Lepidoptera: Noctuidae) and *Anticarsia gemmatalis* Hübner (Lepidoptera: Erebidae) (Pitta et al. 2010; Boiça Júnior et al. 2011), whereas Di Bello et al. (2015) reported non-preference and antibiosis of peanut genotypes against *S. bosqueella*.

Although previous studies have reported non-preference and antibiosis in peanut genotypes against *S.bosqueella* (Di Bello et al. 2015), information on the resistance of currently cultivated commercial peanut cultivars remains limited. Therefore, this study aimed to evaluate possible sources of antibiosis resistance in commercial peanut cultivars to RPW and characterize their relative levels of resistance based on insect biological performance and antibiosis-related parameters.

## Materials and Methods

### Experimental Conditions

The study was conducted at the Laboratório de Resistência de Plantas a Insetos (LARPI), Departamento de Ciências da Produção Agrícola, Faculdade de Ciências Agrárias e Veterinárias (FCAV/UNESP), Jaboticabal, state of São Paulo, Brazil. The bioassay was conducted in the laboratory under ambient controlled conditions of temperature (25 ± 2°C), relative humidity (70 ± 10%), and photoperiod (12 L:12D h).

### Collection of *Stegasta bosqueella* Adults and Egg Production

To obtain first-generation larvae in the laboratory for use in the bioassay, approximately 200 *Stegasta bosqueella* adults were collected using an entomological net in commercial peanut (*Arachis hypogaea* L.) fields in the municipality of Jaboticabal, São Paulo State, Brazil (21°12′28.47''S, 48°17′40.40''W; 603 m altitude). The adults were stored in test tubes (39 cm^3^) sealed with plastic film and transported to the laboratory. The insects were transferred to two glass cages (30 × 30 × 40 cm), with the upper part covered with voile fabric and the bottom lined with filter paper (adapted from Boiça Júnior et al. 2011). The cages were maintained in a climate-controlled room at 25 ± 2°C, 70 ± 10% relative humidity, and a 12-h photoperiod. Four peanut plants (cv. IAC 505), approximately 15 days old, were placed inside each cage as oviposition substrates. After three days, the plants were inspected, and the eggs were collected using a fine paintbrush with soft bristles. The eggs were individually transferred to Petri dishes (9 cm in diameter) lined with moistened filter paper until hatching.

Neonate larvae (< 12 h after hatching) obtained from these eggs were used in the bioassays. Ten neonate larvae were placed in each Petri dish containing leaflets of a single peanut cultivar. Each Petri dish was considered one experimental unit (replicate). Six Petri dishes were prepared for each cultivar, resulting in six replicates per treatment. The biological parameters evaluated, including developmental duration, larval and pupal weight, survival, and fitness index, corresponded to the mean values obtained from each Petri dish and were used in the subsequent statistical analyses.

### Evaluation of the Antibiosis Resistance of Peanut Cultivars on *Stegasta bosqueella* Development

Five commercial peanut cultivars were evaluated for their levels of antibiosis against *Stegasta bosqueella*, namely, IAC Runner 886 (56% oil content), IAC 503 (48% oil content), IAC OL3 (46–47% oil content), IAC Caiapó (59% oil content), and Granoleico (50.61% oil content) (Riveros et al. 2009). During the 2021/2022 agricultural season, IAC cultivars were sown in approximately 80% of the peanut-growing area in the state of São Paulo, with IAC 503 and IAC OL3 accounting for about 60% of the total cultivated area (Chiorato et al. 2022). In addition to the IAC cultivars, the Argentine high-oleic cultivar Granoleico was included because of its distinctive fatty acid profile and relevance to the peanut industry (Nepote et al. 2006). Seeds of these cultivars were sown in 500-mL plastic cups containing substrate composed of soil (eutrophic dark red latosol) (Centurion et al. 1995), sand, and bovine manure (2:1:1) and maintained in a greenhouse (50 mesh anti-aphid screen) until use in the bioassay. The peanut plants were irrigated daily.

We investigated the effects of peanut cultivar on the developmental parameters and survival of RPW via a no-choice bioassay performed in Petri dishes in the laboratory. The bioassay was carried out in a climate-controlled room with a temperature of 25 ± 2°C, 70 ± 10% relative humidity, and a 12 L:12 D h photoperiod. Newly hatched larvae of RPW (< 12 h) were collected with the aid of a wet paintbrush with a fine bristle from the rearing colony and placed individually in Petri dishes (9 cm diameter) lined with moist filter paper. Leaflets were collected from 15-day-old peanut plants of each evaluated cultivar in the greenhouse and offered to RPW larvae in Petri dishes. The bioassay was conducted in a completely randomized design with six replications, in which each replication was composed of 10 larvae individually placed in Petri dishes. For statistical analyses, each Petri dish was considered the experimental unit. Therefore, the values of larval survival, developmental duration, larval weight, pupal weight, and fitness index corresponded to the mean values (or proportion, in the case of larval survival) obtained for each Petri dish.

During the entire larval period, RPW larvae were fed ad libitum with leaflets of the respective peanut cultivars, and the food supply ceased when the insects reached the pupal stage. Leaflets were replenished daily to maintain adequate nutritive quality of food for larval growth. The Petri dishes were periodically cleaned, the feces were removed, and the leaflets were left undisturbed to avoid unintended larval mortality caused by insect disease. After adult emergence, the insects were not provided with food to evaluate only the antibiosis expressed by the peanut cultivars on RPW development.

The survival of RPW larvae was recorded daily, and the mean survival rates on each peanut cultivar were analyzed, accounting for the entire larval period. The duration of the larval period was recorded and analyzed for surviving RPW larvae. The weights of the 7-day-old larvae and 24-h-old pupae were evaluated via a precision analytical scale (0.0001 g) (Ohaus Corporation, AR2140, Parsippany, USA). The pupae were sexed by observing the morphology of the abdomen (Boiça Júnior et al. 2011) with the aid of a stereoscopic microscope at 40 × magnification (Olympus Optical, model SZ-40/SZ-ST, São Paulo, Brazil). To estimate the population growth of *S. bosqueella*, the fitness index (FI), as proposed by Jallow and Zalucki (2003) and adapted by Boregas et al. (2013), was calculated. The FI is based on data on the duration of larval development, pupal weight, and larval survival, which estimates the offspring performance of the tested insects.

Here, the fitness index was calculated as follows: FI = lx.mx/tl; where lx = larval survival, mx = pupal weight, and tl = duration of larval development.

### Statistical Analysis

Data obtained from RPW were checked for normality of residuals and homogeneity of variances via the Kolmogorov‒Smirnov (Kolmogorov 1933; Smirnov 1939) and Levene (Levene 1961) tests, respectively. After confirming the statistical assumption of analysis of variance, the data were subjected to ANOVA, and when significant differences were obtained, the means of the RPW biological parameters of the cultivars were compared via Tukey’s test (α = 0.05) via the PROC GLM procedure. The data for larval survival were subjected to survival analysis by using Kaplan–Meier estimators (log-rank test; α = 0.05; Kaplan and Meier 1958) with PROC LIFTEST. Data from the prepupal period and larval survival did not meet the requirements of ANOVA and were analyzed by the Kruskal‒Wallis test (α = 0.05) via the PROC NPAR1WAY procedure. All the data were analyzed in SAS version 9.4 (Sas Institute 2013).

All biological parameters of* S. bosqueella* were subjected to principal component analysis (PCA) using R software (R Core Team 2026). Subsequently, K-means cluster analysis was applied to the PCA scores to identify groups of observations with similar biological responses. The results were visualized using a PCA biplot, in which the clusters were represented by 95% confidence ellipses and the biological variables by vectors. Graphical representations were generated using the factoextra package.

## Results

**Fig. 1:**
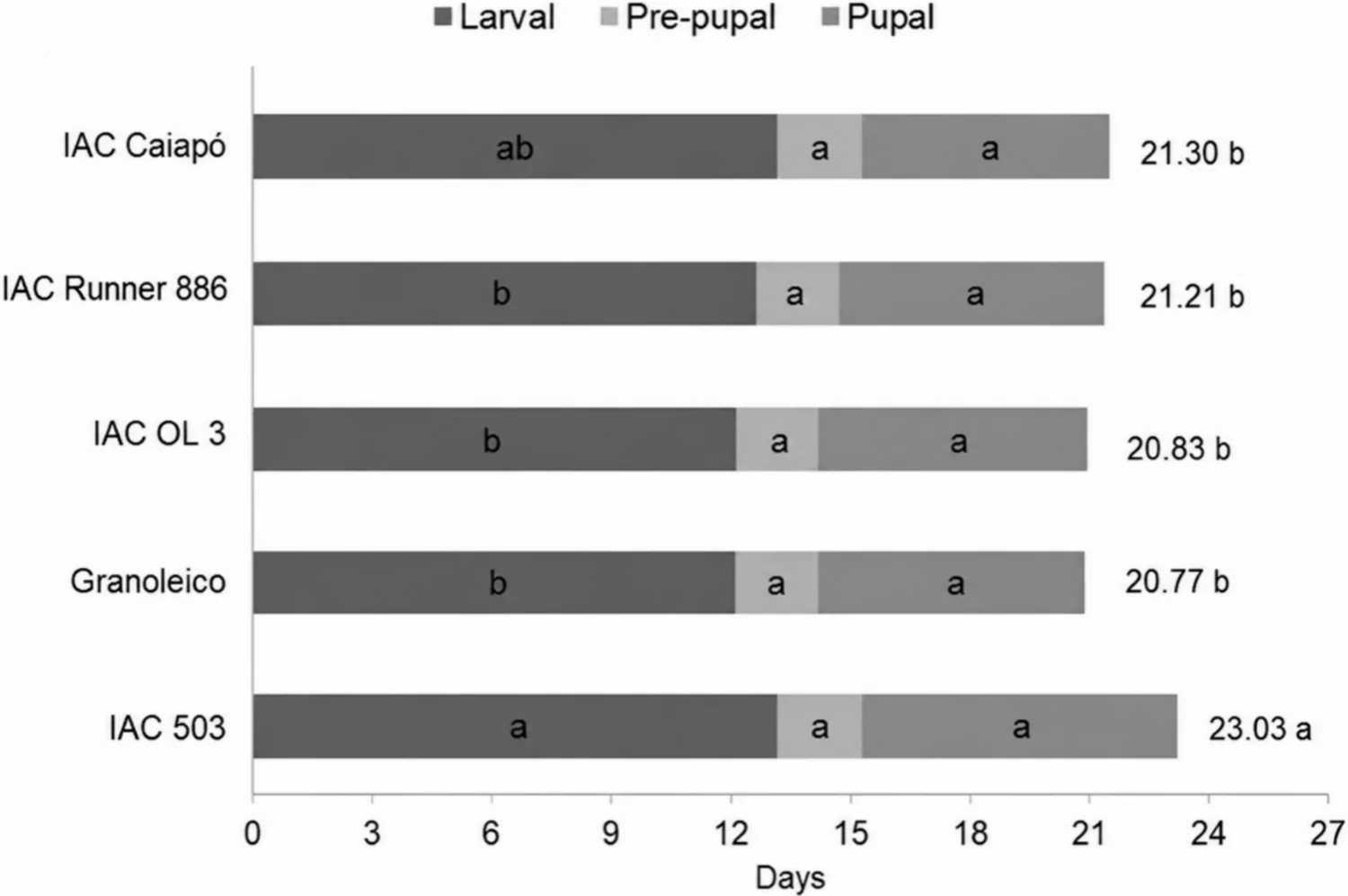
Duration (days) of larval, prepupal, pupal, and total (larvae to adult) periods of *Stegasta bosqueella* fed on peanut cultivars. ^1^Different letters above the bars indicate significant differences (*P* < 0.05)

The total immature developmental period of *S. bosqueella* was significantly affected by peanut cultivar (Fig. 1; Supplementary Table S1). The immature development lasted longer on cv. IAC 503 (23.03 days) than on cvs. Granoleico (20.77 days), IAC OL3 (20.83 days), IAC Runner 886 (21.21 days), and IAC Caiapó (21.30 days) (Fig. 1).

The larval developmental period was also significantly influenced by peanut cultivar (F4,25 = 4.77; *P* = 0.0054) (Fig. 1). Larvae fed leaves of cv. IAC 503 exhibited a longer larval period than those fed cvs. IAC OL3, IAC Runner 886, and Granoleico. The larval developmental period of insects fed cv. IAC Caiapó did not differ significantly from that observed for cv. IAC 503 or the remaining cultivars (Fig. 1).

The durations of the prepupal (H_4, 25_ = 2.56; *P* = 0.40; Fig. 1) and pupal (F_4, 25_ = 1.29; *P* = 0.3036) periods of RPW did not differ significantly among the peanut cultivars (Fig. 1). The mean duration of the prepupal period ranged from 1.32 to 1.57 days, and the duration of the pupal period varied from 6.47 to 6.87 days (Fig. 1).

Larval weight did not differ significantly among peanut cultivars (F₄,₂₅ = 2.61; *P* = 0.0594), although larvae fed leaves of cv. IAC 503 tended to exhibit lower weights than those fed leaves of cv. Granoleico (Table 1). RPW larvae fed leaves of cv. IAC 503 presented pupal weights 1.6- and 1.2-fold lower than those fed leaves of the Granoleico and IAC Caiapó cultivars, respectively. Cultivars IAC Runner 886 and IAC OL3 provided greater pupal weights (F_4, 25_ = 14.98; *P* < 0.0001) than did cv. IAC 503; this cultivar did not differ from IAC Caiapó and Granoleico (Table 1).

**Table 1:** Means (± SE) of larval weight (mg), pupal weight (mg), adult longevity (days), fitness index, and adult emergence (%) of *Stegasta bosqueella* reared on peanut cultivars

Cultivar	Larval weight^1^	Pupal weight¹	Adult longevity^1^ (days)	Fitness index^1,2^	Adult emergence (%)
					♀/♂
IAC Caiapó	3.22 ± 0.41a	3.25 ± 0.08b	2.37 ± 0.14b	15.57 ± 1.94ab	55.00/45.00
IAC Runner 886	3.51 ± 0.39a	3.85 ± 0.29ab	2.25 ± 0.11b	16.17 ± 3.37ab	50.00/50.00
IAC OL 3	3.85 ± 0.37a	3.76 ± 0.11ab	2.50 ± 0.20ab	20.37 ± 2.26a	40.00/60.00
Granoleico	4.13 ± 0.45a	4.04 ± 0.22a	3.26 ± 0.22a	21.30 ± 2.69a	48.00/52.00
IAC 503	2.46 ± 0.35a	2.66 ± 0.09c	2.00 ± 0.22b	6.75 ± 1.30b	47.00/53.00

^1^Means followed by different lowercase letters within a column are significantly different according to Tukey's test (*P* < 0.05)

^2^Fitness index = (lx × mx)/tl, where lx = larval survival, mx = pupal weight (mg), and tl = larval developmental period (days)

Compared with those of cultivars IAC 503, IAC Runner 886, and IAC Caiapó, the longevity of adult RWP of the cutivar Granoleico was greater (F_4, 25_ = 6.58; *P* = 0.0009), whereas that of IAC OL3 did not differ from those of the other peanut cultivars (Table 1).

The fitness index (FI) was 3.2 and threefold lower in RPW larvae fed on cultivar IAC 503 than in those fed cultivars Granoleico and OL3, respectively. In contrast, the FI of larvae fed leaves of IAC Caiapó and IAC Runner 886 did not differ (F_4, 25_ = 2.87; *P* = 0.04) from that of the other cultivars (Table 1). The lowest percentage of emerged females was observed in cv. IAC OL3 (40%) (F_4, 25 =_ 1.18; *P* = 0.3421), while in the other peanut cultivars, the percentage ranged from 47 to 55 (Table 1).

**Fig. 2:**
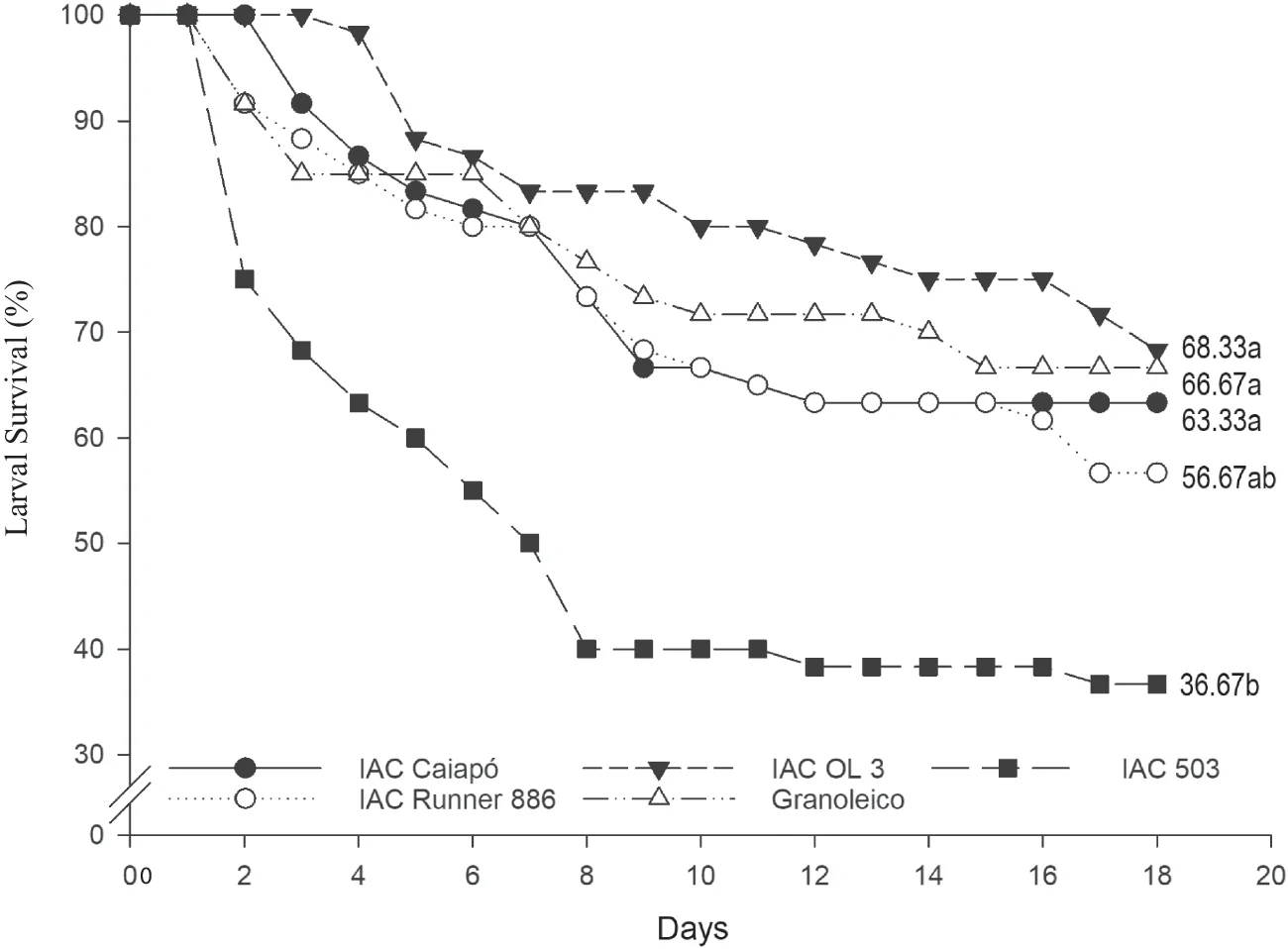
Survival curves of *Stegasta bosqueella* larvae fed different peanut cultivars. Values shown at the end of each curve represent the final larval survival (%). Means followed by different letters differ significantly according to Tukey's test (*P* < 0.05)

The survival curves differed significantly among peanut cultivars (Fig. 2; Supplementary Table S1). Cultivar IAC 503 caused greater larval mortality during the first days of feeding and resulted in the lowest survival at the end of the larval period (H₄,₂₅ = 12.76; *P* = 0.012), whereas IAC Runner 886 exhibited an intermediate survival pattern and did not differ statistically from either IAC 503 or the cultivars IAC OL3, Granoleico, and IAC Caiapó (Fig. 2).

**Fig. 3:**
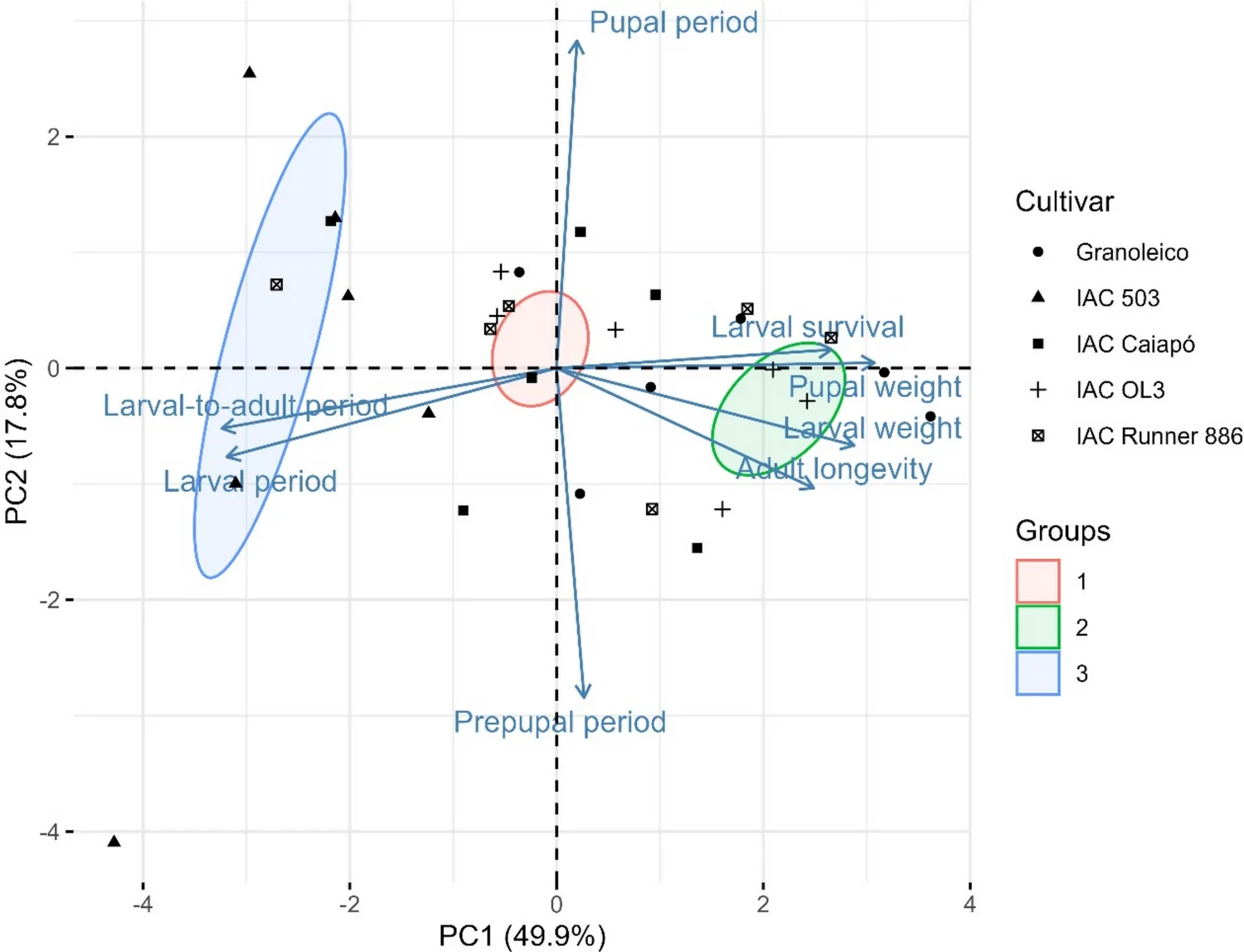
Principal component analysis (PCA) of the biological parameters of *Stegasta bosqueella* fed on different peanut cultivars. The observations were grouped using the K-means clustering algorithm (k = 3), and the resulting clusters are represented by 95% confidence ellipses. Symbols represent peanut cultivars, whereas arrows indicate the contribution of the biological variables to the first two principal components

The principal component analysis (PCA) explained 67.7% of the total data variability, with the first principal component (PC1) accounting for 49.9% and the second principal component (PC2) for 17.8% (Fig. 3). PC1 was primarily responsible for discriminating the observations, separating positive scores associated with larval survival, larval weight, pupal weight, and adult longevity from negative scores associated with larval period and larval-to-adult period, indicating an inverse relationship between developmental duration and biological performance. PC2 was mainly influenced by pupal period and prepupal period, whose vectors were oriented in opposite directions, suggesting a negative correlation between these variables. To facilitate the interpretation of the multivariate structure, the PCA scores were subsequently partitioned using the K-means clustering algorithm (k = 3), and the resulting clusters were represented by 95% confidence ellipses. Cluster 2 was associated with higher values of larval survival, larval weight, pupal weight, and adult longevity, whereas Cluster 3 was associated with longer larval and larval-to-adult developmental periods. Cluster 1 was located near the origin of the ordination space, representing observations with intermediate values for the evaluated biological traits. The distribution of cultivar symbols across the three clusters indicates that the multivariate grouping was not determined by cultivar identity but rather by the similarity of the biological responses measured, as identified by the K-means clustering algorithm.

## Discussion

Peanut crops are attacked by RPW larvae in the major producing regions of Brazil, which are mostly concentrated in areas of the state of São Paulo (Chiorato et al. 2022). This insect species, along with the silvering thrips *Enneothrips flavens* Molton (Thysanoptera: Thripidae), is considered a primary pest of peanuts in the country and elsewhere, causing economic losses in grain production owing to larval defoliation in the youngest leaflets (Pinto et al. 2020a). Thus, it is highly important to carry out studies that evaluate control tactics that could be effective against RPW in the field, such as host plant resistance, with the aim of reducing damage to peanut crops. This study evaluated the development of RPW in selected commercial peanut cultivars that are grown in the producing regions of Brazil and have high productive potential.

The development of RPW larvae that fed on leaves of cv. IAC 503 was negatively affected in most biological parameters. The cultivar IAC 503 extended the larval period of RPW, suggesting the presence of resistance mechanisms in its leaf tissues. According to Santos et al. (2005), when comparing food substrates for insect development, the plant that provides the longest duration in stages of the insect life cycle is considered the worst for its development. The most appropriate plant food sources allow for faster development and greater survival rates (Nava and Parra 2005; Smith 2005). In practical terms, longer developmental periods may result in fewer pest generations during the crop cycle (Dong et al. 2017), slowing population buildup and hence the amount of inflicted injury to plants.

In terms of the weight of RPW immatures (larvae and pupae), cv. IAC 503 provided the lowest weight gains, whereas the Granoleico cultivar presented the highest mean values. This finding suggests that the qualitative and quantitative proportions of primary and secondary compounds ​​that may make up the plant composition of peanut genotypes can affect the immature weight of the RPW. For example, Nogueira et al. (2019) reported that larvae of *Spodoptera frugiperda* (J. E. Smith) (Lepidoptera: Noctuidae) fed on maize leaves of the landrace Pérola or cultivar BRS-Caatingueiro presented the lowest weights, and the resistance of these genotypes was attributed to the absence of nutritional compounds, which reduced the assimilation of ingested food. Santos and Boiça Júnior (2001) and Santos et al. (2005) also reported that the occurrence of low pupal weights indicates insect feeding nonpreference or antibiosis in the plant genotypes that insects were fed. Prolonged larval-to-adult development indicates low nutritional quality of the host plant or the expression of antibiosis (Smith 2005).

For holometabolous insects, food intake and nutrient reserves acquired during the larval stage are highly important because they influence adult dispersal and reproduction (Honěk, 1993; Arrese and Soulages 2010; Parra 2012). In this sense, adult longevity was affected by the cultivars IAC 503, IAC Caiapó, and IAC Runner 886, which reduced the RPW adult life span. Variation in developmental parameters such as survival may have resulted from differences in the nutritional quality of the peanut cultivars, reflected in the availability of essential nutrients required for insect development (Razmjou et al. 2006; Bastos et al. 2007), or from differences in the levels of secondary metabolites (Chen 2008). Several studies have demonstrated the role of secondary metabolites in plant defense, including constitutive and induced resistance to insect attack and pathogen infection (Ryan 1990). These metabolites are generally classified into three major groups: phenolics, terpenoids, and nitrogen-containing compounds (Chen 2008). Future studies should investigate the composition of primary and secondary metabolites in the evaluated peanut cultivars to determine whether these compounds contribute to the resistance mechanisms observed in this study.

To provide an integrated measure of immature performance, Jallow and Zalucki (2003) proposed the biological fitness index (rL), which was later adapted by Boregas et al. (2013). This index integrates larval survival, pupal weight, and larval development time, and is calculated as rL = (lx × mx)/tl, where lx is the larval survival rate, mx is the mean pupal weight, and tl is the larval development period. Higher rL values indicate better host suitability and greater overall performance of immature insects. Boregas et al. (2013) reported a positive correlation between the biological fitness index, pupal weight, larval survival, and larval development period. Therefore, the biological fitness index provides an integrated measure of host suitability based on immature development.

The highest biological fitness index (rL) values were observed for the Granoleico and IAC OL3 cultivars, indicating greater host suitability for *S. bosqueella* under the conditions evaluated. In contrast, the low rL value observed for cv. IAC 503 suggests that this cultivar negatively affected immature development, likely as a consequence of reduced larval survival, lower pupal weight, and a longer larval developmental period, which together reduced the overall biological fitness index. These differences may result from a combination of morphological and chemical plant traits, such as trichome density, tissue hardness, plant structural arrangement and thickness, epicuticular waxes, fiber, cellulose, hemicellulose, and protein content (Veiga et al. 1992; Eigenbrode and Pillai 1998; Pickett et al. 1999; Mallikarjuna et al. 2004; Boiça Júnior et al. 2015; Monteiro 2016).

The high mortality of RPW first instars on cv. IAC 503 suggests that the larvae were affected by resistance mechanisms in this genotype, regardless of the underlying chemical or morphological plant traits, which altered larval feeding behavior (antixenosis) and/or development (antibiosis). Antibiosis is associated with plant characteristics that negatively affect insect growth and development, whereas antixenosis involves plant traits that influence the behavior of larvae and adults in relation to the host plant (Smith 2005). Although antibiosis and antixenosis exert distinct effects on insects, these mechanisms may overlap, particularly during larval development, making it difficult to distinguish between the two categories of resistance (Stout 2013).

Although RPW is considered to be a key defoliator pest of peanut plants, little information exists in the literature concerning the resistance levels of genotypes to insect species. Among the available results, Di Bello et al. (2015) assessed the effects of peanut cultivars with straight and runner growth habits on the feeding preference and development of RPW under laboratory conditions and reported that, unlike in the current study, the cultivar IAC Runner 886 reduced larval survival. Campos et al. (2011) tested peanut cultivars for the expression of antibiosis against *S. frugiperda*, a secondary pest of the crop, and reported that larvae fed leaves of cv. IAC Runner 886 exhibited lower larval survival. Based on these results, cv. IAC Runner 886 was classified as moderately resistant due to antibiosis. In the present study, however, this cultivar did not exhibit the same level of resistance. This difference may be related to the target insect species, as insect responses to plant morphological and chemical traits can vary according to their degree of host specialization.

Other studies have shown that sources of resistance to pests and diseases in wild *Arachis* species are potentially the basis for the selection of resistant progenies, allowing for the development of novel commercial peanut cultivars (Janini et al. 2010; De Paula et al. 2017; Michelotto et al. 2017). According to Michelotto et al. (2017), there are no commercial peanut cultivars that have been genetically modified for resistance to insect pests due to the high costs associated with regulatory processes and cultivar release, in addition to the low genetic efficiency with respect to resistance traits. However, this current situation may no longer be an issue in the near future. According to Smith (2021), conventional plant breeding aimed at resistance to insects will contribute to the global food supply, where projections point to a 30% increase in the global human population by 2050.

The multivariate analysis provided a complementary perspective on the biological responses of *S. bosqueella* to the evaluated peanut cultivars. The PCA, followed by K-means clustering, indicated that the multivariate grouping was primarily determined by similarities among biological traits rather than by cultivar identity. Cluster 2 was associated with greater larval survival, larval weight, pupal weight, and adult longevity, whereas Cluster 3 was associated with prolonged larval and larval-to-adult developmental periods. The overlap of cultivar observations among the clusters indicates that the biological responses were not exclusively associated with a single cultivar but reflected variation among individual experimental units.

Overall, the individual biological parameters consistently indicated that cultivar IAC 503 negatively affected the development of *S. bosqueella*, particularly by prolonging larval development, reducing larval survival, decreasing immature weight, and consequently lowering the biological fitness index. Although the PCA did not separate the cultivars into distinct multivariate groups, the univariate analyses consistently support the presence of antibiosis-related effects in cultivar IAC 503. The use of resistant cultivars is an important component of integrated pest management because it contributes to reducing pest populations, decreasing insecticide applications, and conserving natural enemies. Therefore, the results obtained for IAC 503 highlight its potential as a valuable genetic resource for peanut breeding programs aimed at increasing resistance to RPW. Additionally, farmers may benefit from cultivating this cultivar because (i) it may reduce insect damage, (ii) it has moderate resistance to foliar diseases and relative drought tolerance (Olibone et al. 2021), and (iii) it has shown high productivity under different growing conditions (Fachin et al. 2014). Future studies should investigate the primary and secondary metabolites and other plant traits responsible for the observed antibiosis-related effects, providing a better understanding of the mechanisms underlying resistance.

## Supplementary Information

Below is the link to the electronic supplementary material.

ESM 1 (DOCX 26.8 KB)

## Data Availability

Data will be made available on reasonable request to the corresponding author.
