# Supplementary material for: Development of Stegasta bosqueella (Chambers) (Lepidoptera: Gelechiidae) in Peanut Cultivars
Source: Neotrop Entomol. 2026 Sep 11;55(1):85. doi: 10.1007/s13744-026-01435-1 (PMC13569594; doi:10.1007/s13744-026-01435-1)
Supplement: Supplementary file 1 [file 13744_2026_1435_MOESM1_ESM.docx]

**Supplementary Table S1.** Mean (± SE) developmental periods and larval survival of *Stegasta bosqueella* reared on five peanut cultivars.

| **Cultivar** | **Larval period (days)** | **Prepupal period (days)** | **Pupal period (days)** | **Larval-to-adult period (days)** | **Larval survival (%)** |
| --- | --- | --- | --- | --- | --- |
| IAC Caiapó | 13.28 ± 0.32ab | 1.49 ± 0.16 | 6.54 ± 0.15 | 21.30 ± 0.31b | 66.67± 7.15a |
| IAC Runner 886 | 12.85 ± 0.41b | 1.57 ± 0.09 | 6.70 ± 0.09 | 21.12 ± 0.44b | 56.67± 8.02ab |
| IAC OL3 | 12.79 ± 0.40b | 1.50 ± 0.10 | 6.54 ± 0.11 | 20.82 ± 0.29b | 68.33± 6.00a |
| Granoleico | 12.98 ± 0.34b | 1.32 ± 0.09 | 6.47 ± 0.05 | 20.77 ± 0.32b | 63.33± 4.21a |
| IAC 503 | 14.97 ± 0.57a | 1.50 ± 0.19 | 6.55 ± 0.36 | 23.03 ± 0.45a | 36.67± 5.57b |

Values are presented as mean ± standard error (SE). Each Petri dish containing 10 neonate larvae was considered one experimental unit (n = 6).
